# Draft genome sequence data of the endophytic actinobacterium *Streptomyces justiciae* WPN32, a potential bioactive compounds producer

**DOI:** 10.1016/j.dib.2023.109844

**Published:** 2023-11-26

**Authors:** Montri Yasawong, Wannika Pana, Panjamaphon Chanthasena, Napatsorn Santapan, Thunwarat Songngamsuk, Manassanan Phatcharaharikarn, Phongsakorn Ganta, Supavadee Kerdtoob, Nawarat Nantapong

**Affiliations:** a Programme on Environmental Toxicology, Chulabhorn Graduate Institute, Bangkok 10210, Thailand; b Center of Excellence on Environmental Health and Toxicology (EHT), OPS, MHESI, Bangkok 10400, Thailand; c School of Preclinical Sciences, Institute of Science, Suranaree University of Technology, Nakhon Ratchasima 30000, Thailand; d Faculty of Allied Health Sciences, Nakhon Ratchasima College, Nakhon Ratchasima 30000, Thailand

**Keywords:** Albaflavenone, Endophyte, Inoformatipeptide, Lanthipeptide, Nonribosomal peptide, Polyketide

## Abstract

*Streptomyces justiciae* WPN32 is an endophytic actinobacterium isolated from the rhizosphere of turmeric field soil at the Botanical Garden of Suranaree University of Technology (Nakhon Ratchasima Province, Thailand). Here we present the draft genome sequence of *S. justiciae* WPN32. It was sequenced on the Illumina NextSeq 550 sequencer. The draft genome consisted of 123 contigs with a total size of 9,832,147 base pairs, an N50 of 237,572 base pairs and a GC content of 70.87%. The dDDH between WPN32 and *Streptomyces justiciae* 3R004^T^ was 80.1%, identifying the strain as *Streptomyces justiciae*. The data presented here may aid microbial taxonomy, comparative genomics and identification of gene clusters associated with the synthesis of bioactive compounds. The draft genome sequence data has been deposited at NCBI under Bioproject accession number PRJNA680432.

Specifications Table

**Table:** 

Subject	Biological sciences
Specific subject area	Omics: Genomics
Data format	Raw and analysed
Type of data	Tables, figures
Data collection	Genomic DNA was extracted from a pure culture using the chloroform extraction method and sequenced on an Illumina NextSeq 550. AfterQC v0.9.6 was used for quality assessment, adapter trimming and quality filtering. *De novo* genome assembly was performed using Unicycler v0.5.0 and genome assembly metrics were determined using QUAST v5.0.2. Genome quality was assessed using CheckM v1.1.2. Digital DNA-DNA hybridisations and a phylogenomic tree were analysed using the Type (Strain) Genome Server. Genome annotation was performed using the NCBI Prokaryotic Genome Annotation Pipeline, and potential secondary metabolites were identified by genome mining using antiSMASH v7.0.1.
Data source location	*Streptomyces justiciae* WPN32 was isolated from soil collected from the Botanical Garden of Suranaree University of Technology, Nakhon Ratchasima Province, Thailand at 14.8712º N latitude and 102.0218º E longitude.
Data accessibility	The sequencing data were deposited in the National Center for Biotechnology Information (NCBI) Genbank database under accession number JAVTLL000000000. The deposited draft genome sequencing data are available at https://www.ncbi.nlm.nih.gov/nuccore/JAVTLL000000000.

## 1 Value of the Data

• The draft genome sequence of *S. justiciae* WPN32 could be valuable for research in microbial taxonomy and ecology, in particular for species identification and distribution.
• The draft genome sequence of *S. justiciae* WPN32 has the potential to be beneficial in comparative genomic investigations with other species of *Streptomyces*.
• Elucidating the genome sequence of *S. justiciae* WPN32 could potentially assist in identifying the gene clusters accountable for producing bioactive compounds.

## 2 Data Description

**Fig. 1:**
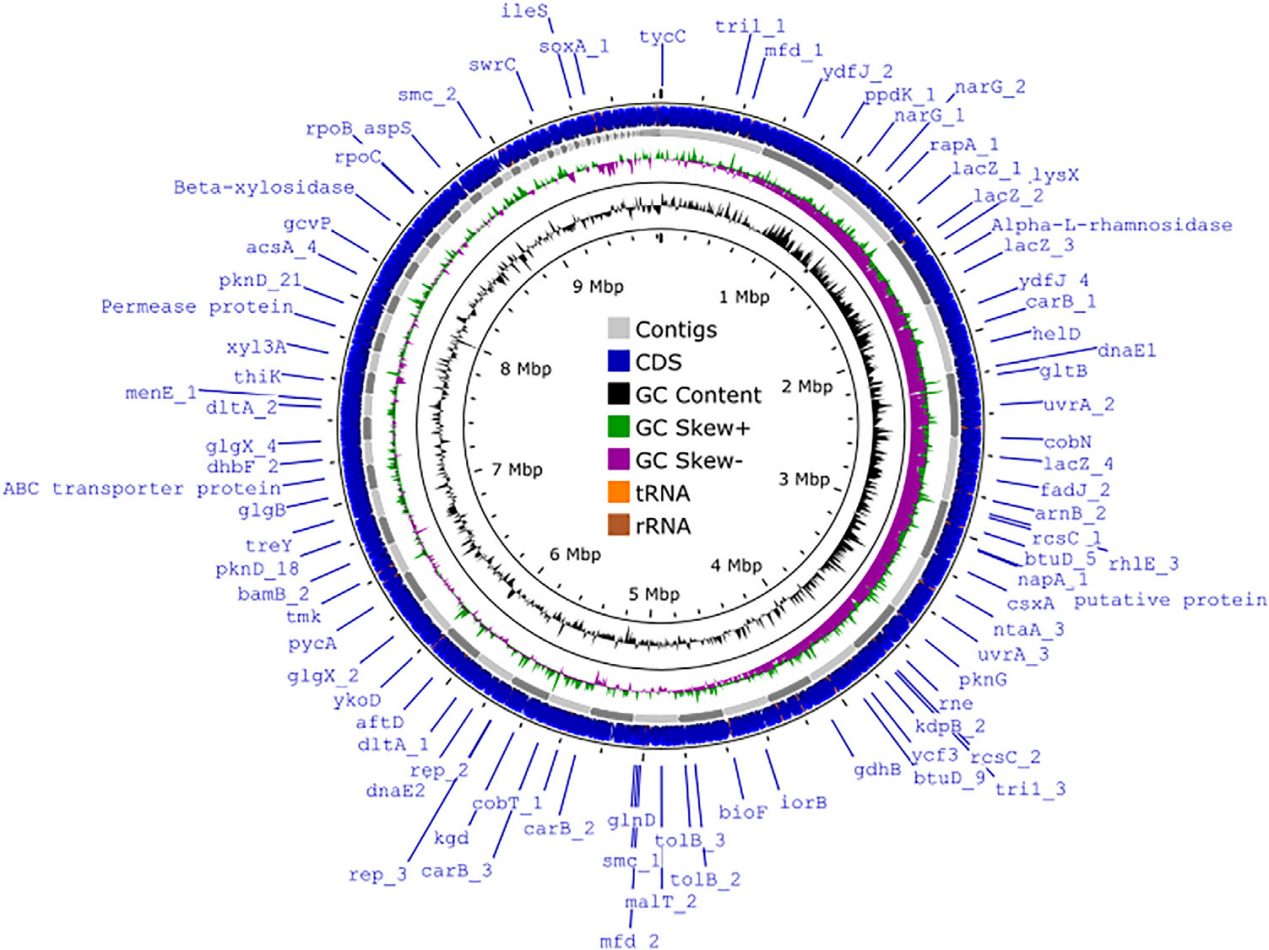
The genome map for *S. justiciae* WPN32 was constructed using the CGView server (https://proksee.ca/, accessed 28 September 2023). CDSs are represented by blue arrows, while contigs are represented by grey arrows. Green peaks represent GC skew+, purple peaks represent GC skew- and black peaks represent GC content.

Here we present the draft genome sequence data of *S. justiciae* WPN32 (Fig. 1), including its gene clusters with potential secondary metabolite biosynthesis.

The genome consisted of 123 contigs with a total size of 9,832,147 bp, an N50 value of 237,572 bp and a GC content of 70.87% (Table 1).

**Table 1:** Genomic features and assembly statistics for *S. justiciae* WPN32.

Attribute	*S. justiciae* WPN32
Genome size (bp)	9,832,147
Number of contigs	123
Genome coverage	135×
GC content (%)	70.87
Largest contig (bp)	554,487
N50 (bp)	237,572
N75 (bp)	127,154
L50	15
L75	28
Total gene	9,096
Total CDS	9,019
tRNA	70
rRNA	4
ncRNA	3

**Fig. 2:**
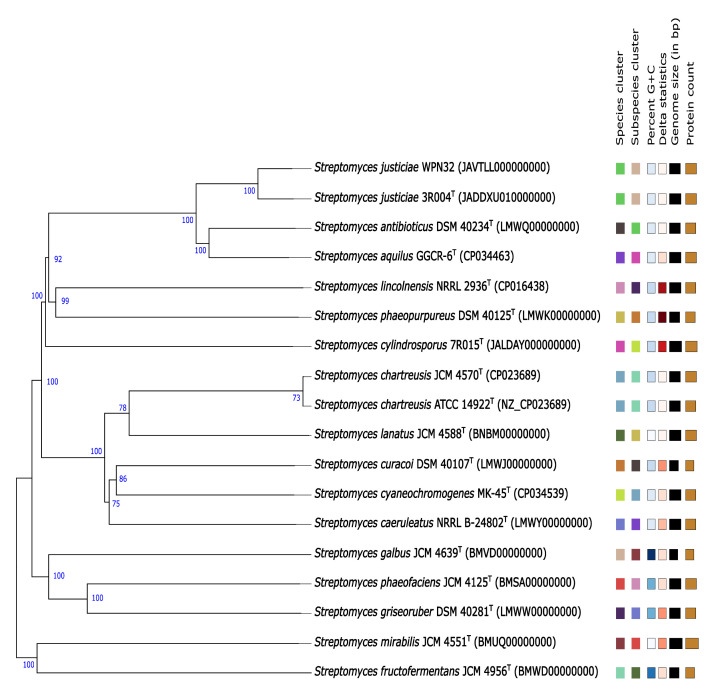
The phylogenomic tree was reconstructed using the whole-genome sequence data of *S. justiciae* WPN32 and its closely related type strain on the TYGS platform. Branch numbers were determined based on pseudo-bootstrap support values greater than 70% from 100 replicates using Genome Blast Distance Phylogeny (GBDP), with average branch support at 93.6%.

The WPN32 draft genome was determined to be 99.14% complete with an estimated contamination of less than 1%. The digital DNA-DNA hybridisation (dDDH) value between WPN32 and *Streptomyces justiciae* 3R004^T^ was 80.1%, indicating that WPN32 is a *Streptomyces justiciae* strain. The phylogenomic tree of the strain WPN32 and closely related type strains is shown in Fig. 2. Genome analysis revealed the existence of recognised biosynthetic categories with significant similarity, namely lanthipeptides (100%), non-ribosomal peptides (NRP) (71-100%), polyketides (93-100%) and terpenes (100%) within the WPN32 genome (Table 2).

**Table 2:** Secondary metabolite biosynthesis gene clusters identified by antiSMASH.

Contig	Type	Position (bp)	Closest known biosynthetic class	Predicted secondary metabolite	Similarity
4	RiPP-like	351,126-378,443	Lanthipeptide	Informatipeptin	100%
5	T2PKS	312,369-381,783	Polyketide	Setomimycin	93%
18	Terpene	149,172-170,257	Terpene	Albaflavenone	100%
19	NAPAA	158,922-192,773	NRP	ε-Poly-L-lysine	100%
24	T3PKS	92,466-133,527	Polyketide	Flaviolin 1,3,6,8-tetrahydroxynaphthalene	100%
65	Other	1-17,306	NRP	Actinomycin D	71%

The analysis indicates that the WPN32 strain has the potential for synthesising various antimicrobial agents, including albaflavenone [1], ε-Poly-L-lysine [2], informatipeptin [3], and setomimycin [4] (Table 2).

RiPP-like, other unspecified ribosomally synthesised and post-translationally modified peptide product (RiPP); T2PKS, type II polyketide synthases; NAPAA, non-alpha poly-amino acids like e-Polylysin; NRP, non-ribosomal peptide; and T3PKS, type III polyketide synthases.

Additionally, the study also demonstrates that WPN32 has potential to produce actinomycin D, a recognized anticancer agent [5], as well as flaviolin 1,3,6,8-tetrahydroxynaphthalene, which exhibits properties of UV protection [6]. We believe that the draft genome sequence will facilitate the characterisation of genes involved in the synthesis of bioactive compounds and the production of secondary metabolites in *S. justiciae* WPN32.

## 3 Experimental Design, Materials and Methods

### 3.1 Bacterial Isolation

Strain WPN32 was isolated from the rhizosphere of turmeric field soil collected at Suranaree University of Technology, Nakhon Ratchasima Province, Thailand (14.8712º N, 102.0218º E). One gram of soil sample was suspended in 99 mL of sterile water in a 250 mL Erlenmeyer flask. The soil suspension was serially diluted and spread on International *Streptomyces* Project-2 (ISP-2) agar. Plates were incubated at 37°C for 5 days or until streptomyces colonies appeared. A single colony of strain WPN32 was selected and purified by cross-streaking onto a new ISP-2 plate.

### 3.2 Genomic DNA Preparation

Genomic DNA (gDNA) was extracted from overnight cultures of strain WPN32 on ISP-2 medium. The cell pellet of the WPN32 strain was mixed with 50 µL of lysozyme (20 mg/mL) and 25 µL of RNase A (5 mg/mL). The mixture was incubated at 37°C for 60 min, then 60 µL of 10% sodium dodecyl sulphate (SDS) and 80 µL of 500 µg/mL Proteinase K were added to the tube and incubated at 50°C for 20 min. The suspension was mixed with 100 µL of 5M NaCl and centrifuged at 7,000 rpm for 15 min. The extraction of gDNA involved the combination of equal proportions of chloroform and isoamyl alcohol. Subsequently, the gDNA was recovered by adding an amount of 95% ethanol equal to twice its volume and placed in an incubator at -80°C for 30 min. Following this, the mixture was centrifuged at 7,000 rpm for 15 min to obtain the gDNA. The supernatant was discarded, and the gDNA underwent three rounds of washing using a 70% ethanol solution. The gDNA pellet was air-dried before resuspension in 30 µL of TE buffer. The gDNA was subsequently evaluated for quality using agarose gel electrophoresis and NanoDrop spectrophotometry (Thermo Scientific, USA).

### 3.3 Whole Genome Sequencing and Assembly

Sequencing libraries were generated from 1 ng of DNA using the Nextera XT DNA library preparation kit (Illumina, San Diego, CA, USA). Raw sequencing reads were obtained through the NextSeq 550 sequencer by employing the NextSeq 500/550 high output kit v2.5 (300 cycles, 2 × 150-bp reads) (Illumina). AfterQC v0.9.6 with default parameters [7] was used for quality assessments, adapter trimming, and quality filtering. *De novo* genome assembly was conducted using the raw reads and Unicycler v0.5.0 under default settings [8]. Genome assembly metrics were determined using QUAST v5.0.2 with default parameters [9].

### 3.4 Taxonomic Identification of the Strain

Genome quality was evaluated through CheckM v1.1.2 with default settings [10]. Digital DNA-DNA hybridisation (dDDH) and a phylogenomic tree, which were produced from the whole genome sequences of WPN32 and related strains, were analysed using the Type (Strain) Genome Server (TYGS) [11].

### 3.5 Genome Annotation and Sequence Analysis

Genome annotation was conducted using the NCBI Prokaryotic Genome Annotation Pipeline (PGAP) with default settings [12]. In addition, potential secondary metabolites were identified through genome mining via antiSMASH v7.0.1 with default settings [13].

## Limitations

Next-generation sequencing techniques generate significant amounts of data, but the *de novo* genome assemblies based on these data are often conspicuously incomplete. These faulty assemblies are prone to resulting annotation errors, most notably in an inaccurate count of genes that may be present within the draft genome of WPN32.

## Ethics Statement

This work does not involve human or animal subjects and the authors declare that this manuscript is original and has not been published elsewhere.

## CRediT authorship contribution statement

**Montri Yasawong:** Methodology, Data curation, Writing – original draft, Writing – review & editing. **Wannika Pana:** Methodology, Data curation. **Panjamaphon Chanthasena:** Methodology, Data curation. **Napatsorn Santapan:** Methodology, Data curation. **Thunwarat Songngamsuk:** Methodology, Data curation. **Manassanan Phatcharaharikarn:** Methodology, Data curation. **Phongsakorn Ganta:** Methodology, Data curation. **Supavadee Kerdtoob:** Methodology, Data curation. **Nawarat Nantapong:** Conceptualization, Data curation, Supervision, Writing – original draft, Writing – review & editing.

## Data Availability

Streptomyces justiciae strain WPN32, whole genome shotgun sequencing project (Original data) (GenBank NCBI).
